# Direct Printing of Nanostructured Holograms on Consumable Substrates

**DOI:** 10.1021/acsnano.0c02438

**Published:** 2021-02-01

**Authors:** Bader AlQattan, Joelle Doocey, Murad Ali, Israr Ahmed, Ahmed E. Salih, Fahad Alam, Magdalena Bajgrowicz-Cieslak, Ali K. Yetisen, Mohamed Elsherif, Haider Butt

**Affiliations:** † Nanotechnology Laboratory, School of Engineering, University of Birmingham, Birmingham B15 2TT, United Kingdom; ‡ Department of Mechanical Engineering, Khalifa University of Science and Technology, P.O. Box 127788, Abu Dhabi, United Arab Emirates; § Manufacturing Group, University of Warwick, Coventry CV4 7AL, United Kingdom; ∥ Department of Chemical Engineering, Imperial College London, London SW7 2AZ, United Kingdom

**Keywords:** holograms, diffraction, nanopatterns, holographic laser ablation, laser interference patterning

## Abstract

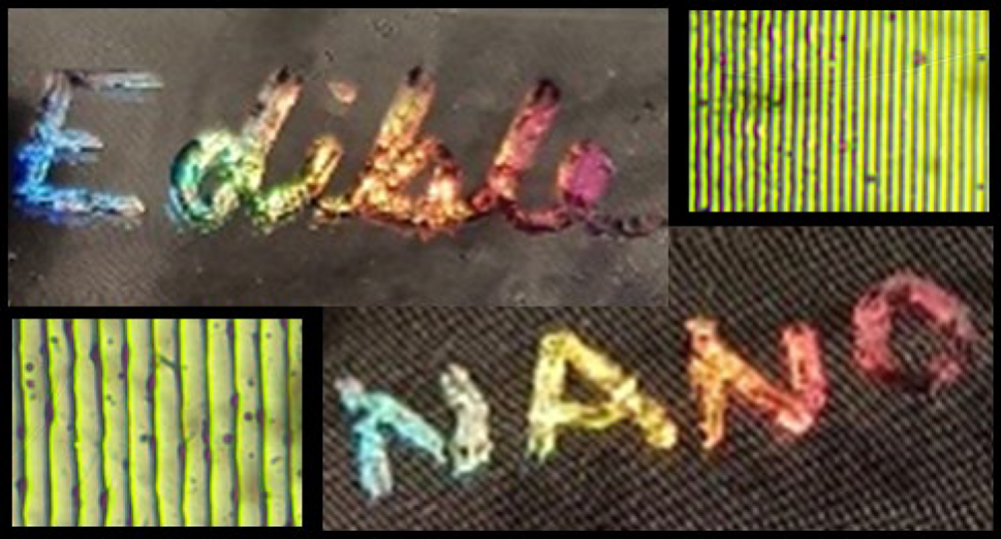

Direct texturing of nanostructures on consumable substrates and products is a challenge because of incompatible ingredients and materials’ properties. Here, we developed a direct laser-based method to print nanostructured holograms on dried films of consumable corn syrup solutions. A holographic laser (λ = 1050 nm) interference system was used to construct the nanostructures of the holograms on food for rainbow effects. The relationship between wavelength and periodicity contributed to the changing diffraction angle through the change of the refractive index (1.642). Increasing the sugar concentration (25–175 mg) in the syrup increased the diffraction efficiency of these holograms. The added amount of sugar in the composition increased the refractive index (7%) and decreased the light absorption (12.9%), which influenced the change of diffraction angle by 4.4°. The surface holograms displayed wideband visual diffraction of light extending from violet to red wavelengths. These holograms on edible materials can be imprinted onto commercial food products for adding aesthetic value and controlling perception.

The use of holograms in food could potentially improve sensory appeal and, through biosensing, could increase health and safety.^1,2^ Holograms can even be used to store information as edible microtags.^3^ They are also attractive to the eye as they produce rainbow patterns with light. Using edible holograms on foods, not only as decoration but also to sense harmful bacteria, could improve food quality/lifetime monitoring.^4,5^ Food holograms which signify a qualitative information about the sugar contents could be of value in controlling the sugar consumption, that is challenging to be measured at the moment.^6^ The potential uses of nanotechnology in the food industry have been steadily increasing.^7^ There was a suggestion of using a CO_2_ laser in the food industry to peel fruits and vegetables.^8^ There are also many applications for using nanoparticles in the food industry, including the following: food packaging and security; improving food functionality by preventing deterioration, and food safety for the consumer.^9^ Additionally, there have even been discussions about the possibility of using nanotechnology to create portable devices for analyzing foods and detecting harmful properties.^10^ Moreover, security holograms have been reported to be used on household food items.^11^ However, using nanoparticles can lead to generation of reactive oxygen species (ROS) (a nanoparticle associated toxicity) or oxidative stress, meaning a screening method is vital as a preventative measure against these nanoparticle-induced toxicities.^12^ It can be a laborious process to get approval for using nanotechnology in food because of standards and regulations from agencies, such as the U.S. Food and Drug Administration (FDA).^13^ Also, research suggests consumers can be reluctant to accept nanotechnology in the food market because of growing concerns associated with using nanostructures and nanoparticles in food industries.^9,14^ However, as applications for nanotechnology are developed and it becomes more widely used, regulations will adapt to allow nanotechnology in the industry, thus giving consumers the confidence that if the food is on the market it has passed the relevant safety tests.^13^ In the future, FDA clearance could allow manufacturers to use ultraviolet (UV) or electron beam sources to “cured inks, coatings, and adhesives”, for producing nanoparticle holograms.^15^

Historically dichromated gelatin (DCG) has a long history of being used in making holograms.^16^ Hardened dichromated gelatin is considered as one of the known materials today for the fabrication of holographic optical elements.^17^ These hardened DCG holograms are formed by exposing the gelatin to actinic radiation then removing residual chemical compounds in a development stage.^17^ Similarly, a simplified production method was developed to create holograms in hardened DCG. In this case, gelatin is hardened by soaking in ammonium dichromate to produce holograms recorded at a wavelength of 488 nm and with 90% diffraction efficiency.^18^ More recently, different forms of gelatin have been contemplated for application in diffraction optics.^19^

Computer-generated holograms were produced on sugar (sucrose) crystals by UV microlithography techniques (λ = 240 nm), with a diffraction efficiency of around 45%.^20^ Sugar concentration could be measured in aqueous solutions through the spacing of an optical fiber.^10^ Corn syrup has also been used to create holograms by using thin layers with potassium dichromate solution. It was photosensitized with He–Ne laser (λ = 530 nm) to create holograms with 4% diffraction efficiency.^21^ However, there are some indications that potassium dichromate may be toxic to human or animal health, rendering the holograms inedible.^22^ Therefore, a lithographic rubbing technique was used to create phase or amplitude relief holograms in corn syrup, with a diffraction efficiency of 8.4% when using an amplitude mask or 36% when using a relief mask, all without the use of any photosensitive salts or dyes.^23^ Moreover, many different nanomanufacturing techniques can be used to create holographic interference patterns. E-beam lithography was used to create computer-generated holograms.^24^ Additionally, phase controlled holographic lithography was conducted with a He–Ne laser (λ = 532 nm) to create holograms on liquid crystals.^25^ Laser interference lithography can also create many different periodic structures depending on how many interference beams are used.^26,27^ Carbon nanotube scattering can also produce holograms.^28^ However, these methods can be complicated and require multiple stages, including pre and post baking and using solutions such as acetone to dissolve the resist layer.^29^

In this work, a holographic direct laser interference pattering (DLIP) method was developed to pattern holograms on edible materials. Holographic DLIP is direct, simple, low cost and rapid technique requiring only one processing step in comparison to conventional methods.^30^ This repeatable method is also preferable as it does not require masks or templates and can create periodic patterns covering a large area in a range of sizes.^31^ Additionally, it has been used on the surfaces of many different materials from metals to ceramics.^32^ The holographic DLIP method was developed to pattern one-dimensional (1D) nanostructures on edible substrates. Multiple periodic structures were produced to study the effect of the periodicity. Angle-resolved spectral measurements were performed to characterize their optical properties. The nanostructures were studied to determine their light diffraction properties in response to monochromatic and broadband white light. The influence of increasing sugar concentration in the edible solutions was analyzed to determine the optimal fabrication parameters.

## Results and Discussion

### Fabrication of Nanostructures using Edible Materials

Dried films of corn syrup were used as substrate for imprinting holograms by using the DLIP method, which is the chosen method as it is a simple and swift process as compared with other methods of nanomanufacturing.^33^ Corn syrup was made from a blend of sugars gained from corn-starch (15–20% glucose). Some “light” varieties contain vanilla flavoring, and other “dark” varieties do not. The dark coloring and flavors are similar to molasses.^34^ A “light” corn syrup was used in this work and the effects of adding extra vanilla extract to the corn syrup solution were explored. The samples were left in room temperature for 3 h until they fully dried. The thickness of each sample was of the order of 100 μm. Different solutions were prepared and the samples with optimum properties were selected, with a composition of 1.5 mL of vanilla extract, 2 mL of corn syrup, and 0.5 mL of water (Supporting Information, Table S1). This composition was selected for its superior mechanical strength and hardness at room temperature as compared with other solutions.

**Figure 1:**
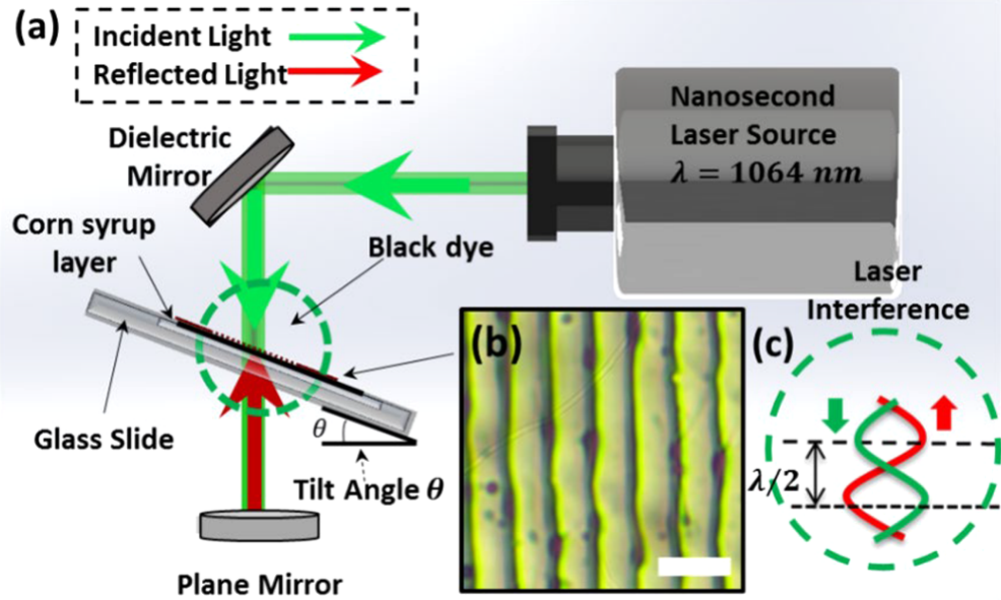
“Denisyuk” reflection interference laser ablation setup. (a) Laser setup schematic for producing holograms on edible thin films using a nanosecond laser with wavelength λ = 1064 nm. (b) Holographic grating produced on dried corn syrup solution. (c) Laser interference causing contrasting areas of high and low intensity laser energy causing the production of holographic grating on thin film surfaces. Scale bar: 5 μm.

A thin layer of synthetic black dye was deposited (900 nm thickness) onto the corn syrup thin films of each sample (Supporting Information, Table S2).^35^ The composition of edible materials and the thickness of the black dye was optimized to achieve absorbance properties for laser ablation (Supporting Information, Table S1 and Figure S1). This dye was chosen because it was competent in maximizing the laser pulse absorption, which enabled the ablation process to generate low-cost nanostructures on the surface of the corn syrup film. Figure 1a shows a schematic of the hologram recording setup in Denisyuk ablation mode. The laser beams (λ = 1064 nm, the laser energy from 210 mJ, 3.5 ns) initially directed by a mirror traveled to the black dye on the corn syrup thin film (recording medium) and reflected off a plane mirror, placed below, to ablate the localized regions on the medium.

The laser interference pattern that causes the periodic structure (Figure 1b) is due to the beam reflected off the plane mirror and the incident beam coinciding with it to form bright and dark fringes (nanopatterning structure).^8,36^ This laser interference forms a standing wave, creating periodic constructive interference (Figure 1c) fringes which ablate the thin film into a periodic nanostructure with dimensions related to the wavelength of the laser and the sample tilt angle.^33,35^ The periodicity of the two interfered laser waves can be described by
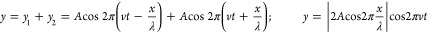
1where *y*_1_, *y*_2_, *A*, *v*, *t*, and λ indicate incident laser beam propagation, reflected laser beam propagation, amplitude, velocity, time, and wavelength. This periodicity corresponds to the constructive interference peaks occurring at approximately λ/2.^37,38^ The tilt angle as the sample is tilted from the horizontal also has an effect on the periodicity of the structure produced. To analyze the relationship between the periodicity and diffraction angle, the nanostructures were observed under a microscope (eq 2). This relationship is given by
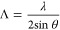
2where Λ, λ, and θ represent periodicity of the nanogratings, wavelength, and tilt angle.^38^ This relationship was utilized in the experiments to create holographic structures of varying sizes by varying the tilt angle. The periodicity of each sample was measured using the optical microscope, and therefore, the relationship between the two parameters could be defined. Optical transmission analysis (normal incidence) was conducted on the nanostructured holograms produced on the corn syrup films.

**Figure 2:**
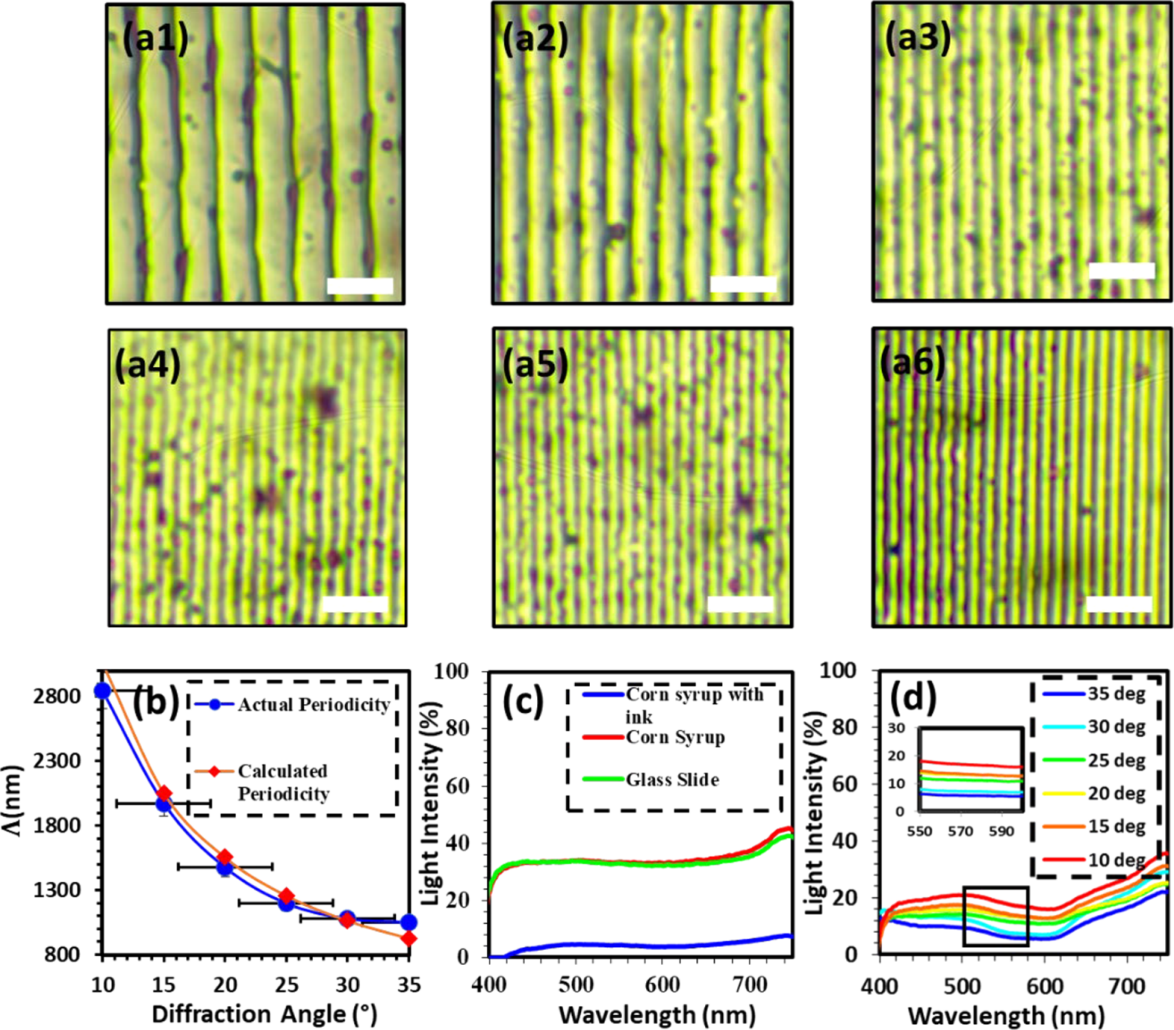
Periodic gratings produced on the corn syrup thin films observed under the optical microscope. (a1–a6) Samples produced at tilt angles: 10°, 15°, 20°, 25°, 30°, 35°. (b) Tilt angle variation with periodicity, corresponding to (a1–a6). (c) Optical transmission spectra comparing plain corn syrup thin film, black ink coated thin film, and plain glass slide. (d) Optical transmission for varying tilt angles; legend represents the tilt angle. Scale bar = 5 μm.

The holographic nanopatterns were produced on top of the corn syrup films, and uniform morphology was observed under the optical microscope (Figure 2a1–a6). These images were used to calculate the periodicity of the nanogratings. Table 1 shows the periodicity ranging from 2850–1050 nm as the tilt angle is changed from 10–35°, and Figure 2b shows a graph of this data compared with the theoretical periodicity (eq 2). The average difference between the graphs are about 3%, validating that periodicity of diffraction gratings is a function of tilt angle.^38^Figure 2c shows the change in optical transmission in between the glass slide (with no corn syrup), plain corn syrup thin film, and with ink coating. Interestingly, the optical transmission of the corn syrup thin film and glass slide are very similar because of the transparency of the corn syrup solution. In the previous studies, researchers created ink holograms onto glass slides,^39^ and this similarity in between the optical transmission between the glass slides and the corn syrup films gave an indication of a positive response with our holographic DLIP system.^33^ Otherwise, the nanopattering of the hologram would not be successful on the corn syrup films.^35^ When comparing (Figures 2c,d), the optical transmission of the black dye quoted corn syrup film was <5%, whereas after laser ablation (Figure 4d), for all tilt angles, the transmission of the material increased by up to 20%. This was less than plain corn syrup, which has an optical transmission of about 30%. This proves that after ablation, a significant portion of the black dye gets removed from the corn syrup films.

**Table 1:** Results for Varying Tilt Angles and the Comparison between Calculated and Experimentally Obtained Periodicities of the Nanostructures^a^

sample	tilt angle, θ (deg)	actual periodicity (nm)	calculated periodicity (nm)
1	10	2850	3063
2	15	1970	2055
3	20	1480	1555
4	25	1200	1258
5	30	1080	1064
6	35	1050	927
**X®**	22.5	1605	1654
**σ̅**	9.4	699.5	788.7

a **X** average, **σ**: standard deviation.

Because of the mechanical strength of the corn syrup holograms, their optical transmission analysis could be conducted (Figure 2d). The transmission, and thus absorption, of the material affects the properties of the resulting periodic grating as per to the relationship: absorption % = 1 – transmission %.^39^ Absorption gives an indication of the laser light interaction with the material. Increasing the absorption would contribute toward improving the ablation efficiency with the Nd:YAG laser beam, enhancing the interaction of laser light with the material. The highlighted region between 540 nm and 620 nm shows that by increasing the tilt angle from 10–35°, the transmission decreases from approximately 20% to 5%. The reduced light transmission with changing tilt angle could be due to the increase in absorption due to more unablated ink material on the thin film. At larger title angles, smaller sized nanogratings are produced, which have higher diffraction angles. The large diffraction angles also result in lower optical transmission recorded in normal incidence.^39,40^

**Figure 3:**
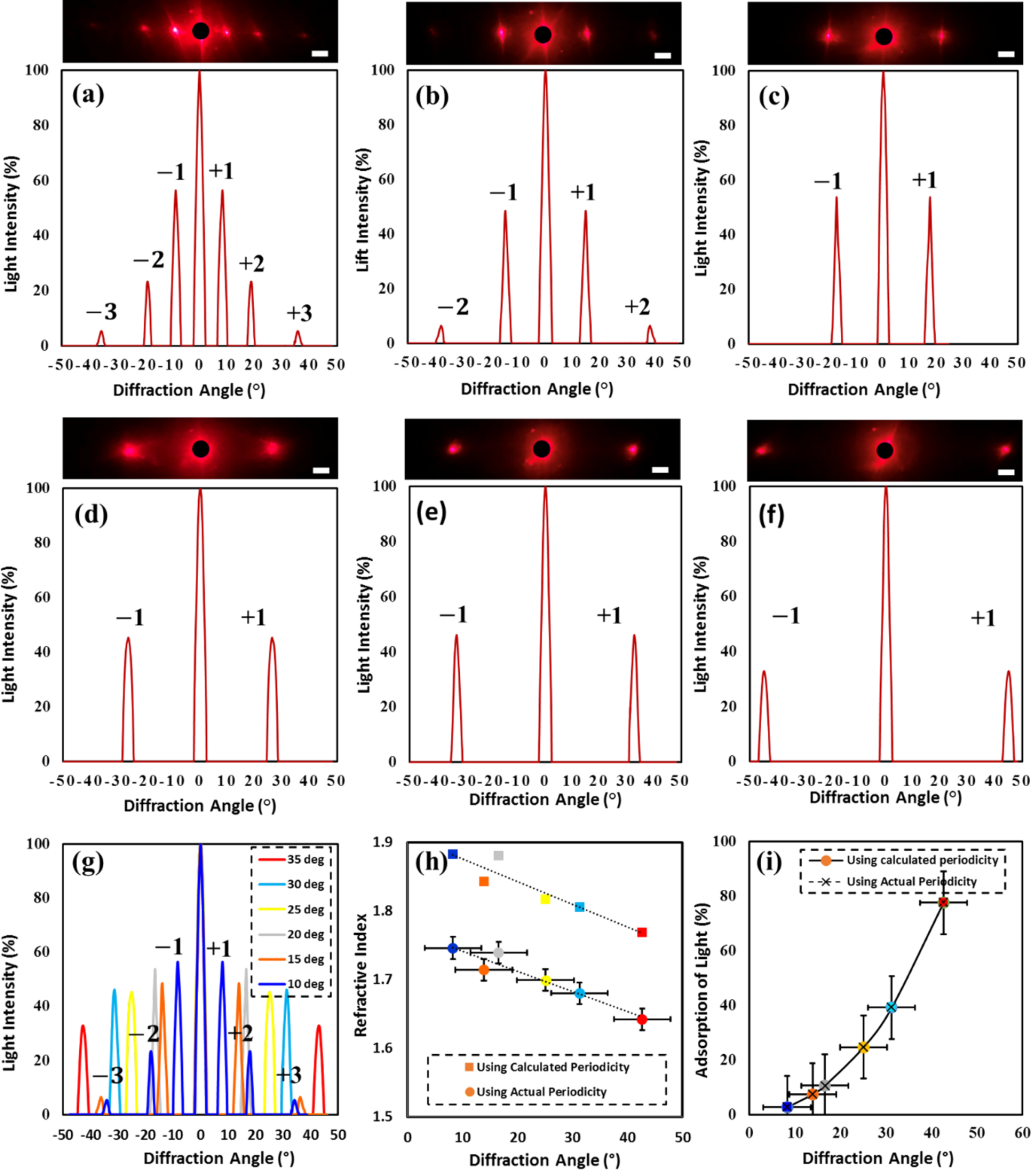
Diffraction produced from nanostructure holograms on edible substrates when illuminated with monochromatic red laser light of wavelength 635 nm. (a–f) Tilt angle: 10°, 15°, 20°, 25°, 30°, 35° respectively. (g) Combined graph of diffraction angle vs intensity showing tilt angles from 10–35°. First-, second-, and third-order diffraction peaks are highlighted. (h) Graph of refractive index against diffraction angle, comparing calculated and actual periodicity used in calculations. (i) Graph of absorption of light against diffraction angle, comparing calculated and actual periodicity used in calculations. Scale bar = 5 cm.

Diffraction analysis was performed to measure the effectiveness of the optical holograms produced. The relationship between the light passing through the gaps, in this case the holographic grating structure, and the positioning of the diffracted light was analyzed. The angle at which the light is diffracted increases when the grating’s period size decreases. Therefore, a relationship between the diffraction of light for each sample and the periodicity can be established to determine the optimum tilt angle. By validating the experimental results with theory, the concept was proved to be effective. The optical diffracting produced by the holographic nanostructure in response to monochromatic red light (Figure 3a–f) shows normalized intensity of light against diffraction angle for the changing sample periodicities. Figure 3g combines the graphs for comparison; the diffraction angles have a large range from 8.29° for sample “a” (tilt angle = 10°) to 42.7° for sample “f” (tilt angle = 35°), showing there is a proportionality relationship between tilt angle and diffraction angle (eq 2).^38^ The intensity of diffraction also reduces with increasing diffraction angle. This is reflected in the transmission analysis (Figure 2d); a decrease in the optical transmission corresponds to the reduction of the structure size. Figures 3h plots refractive index against diffraction angle when using red laser light and when changing the wavelength. In both cases, calculated and experimental results had the same trend, and diffraction angle is inversely proportional to refractive index. Using diffraction analysis, the refractive index and absorption of light can be calculated, by rearranging eqs 3–5:
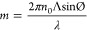
3
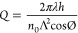
4
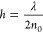
5where *m*, *n*_0_, Λ, Ø, Q, and *h* are diffraction order, refractive index, periodicity, diffraction angle, absorption of light, and grating thickness.^39,42^ As eqs 3 and 4 show, the wavelength of light and periodicity of the grating are affected by both the refractive index and absorption parameters. Therefore, diffraction experiments were conducted to see the effects of changing wavelength and periodicity. These equations can be used to determine if efficiency as absorption of light is related to ablation efficiency of the laser beam: holograms with a low absorption have the least efficiency (Table 2).^35^

**Table 2:** Intensity, Diffraction Angle, Calculated Refractive Index, and Absorption of Light at First-Order Diffraction Peaks for Samples 1–6

sample	max normalized intensity at first-order diffraction	diffraction angle at first-order diffraction (deg)	refractive index, *n*_0_ (experimental periodicity)	absorption of light, *Q* (experimental periodicity)	refractive index, *n*_0_ (calculated periodicity)	absorption of light, *Q* (calculated periodicity)
1	56.5	8.29	1.746	2.61	1.883	2.61
2	48.5	13.9	1.714	7.37	1.843	7.37
3	53.8	16.6	1.739	10.6	1.881	10.6
4	45.4	25.1	1.699	24.6	1.817	24.6
5	46.2	31.3	1.680	39.2	1.806	39.2
6	32.9	42.7	1.642	77.6	1.769	77.6

**Figure 4:**
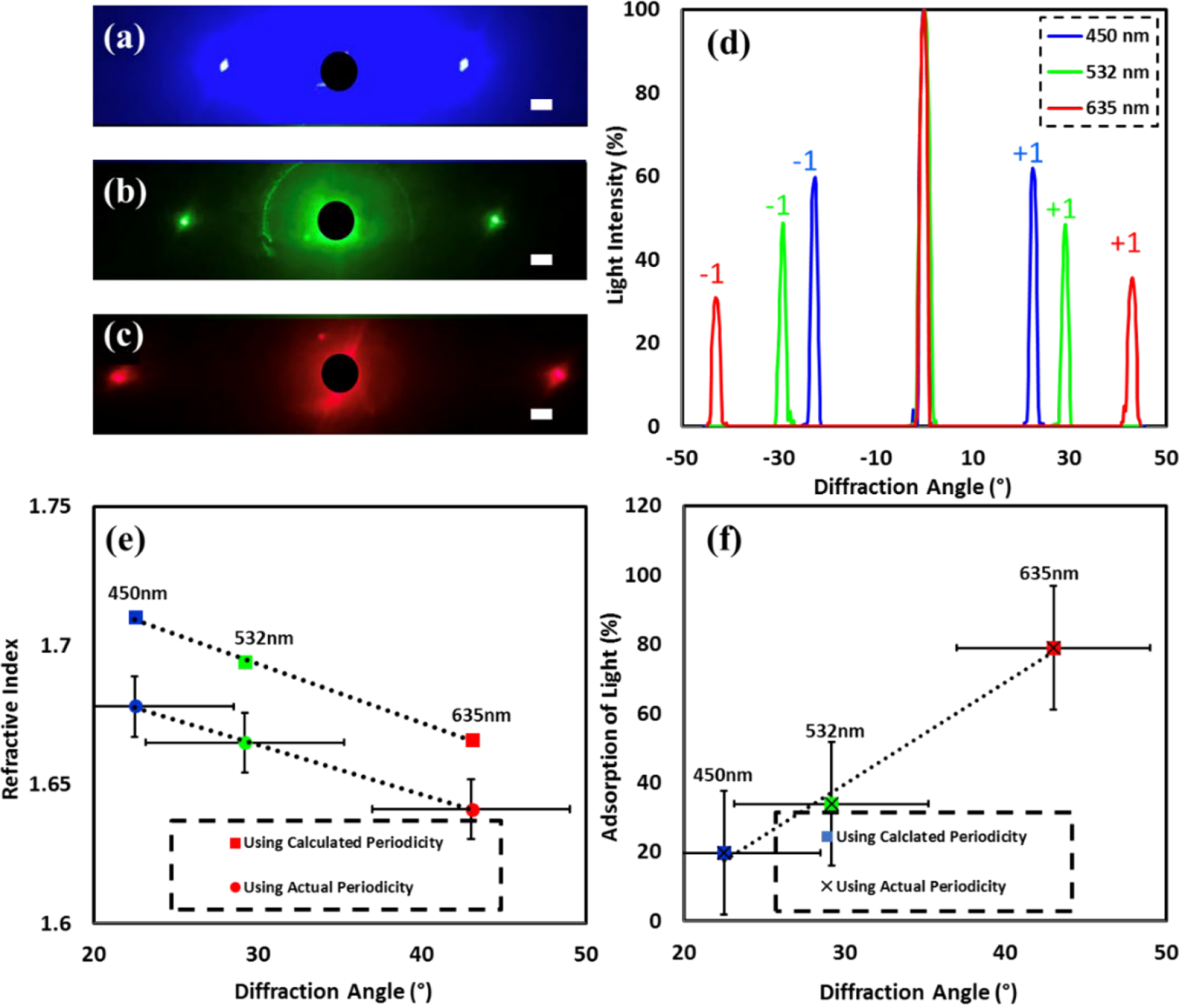
Diffraction produced from nanostructures holograms (1050 nm) when illuminated with monochromatic light of varying wavelength. (a–c) Diffraction patterns produced when nanostructures were illuminated with monochromatic light of wavelength 450 nm (blue), 532 nm (red), 635 nm (green), respectively. (d) Diffraction angle vs normalized light intensity graph comparing different wavelengths; graph color corresponds to laser wavelength, respectively. (e,f) Graph of refractive index and absorption of light respectively against diffraction angle, comparing calculated and actual periodicity used in calculations for all three wavelengths; colors of points correspond to laser wavelength. Scale bar = 5 cm.

Figure 4 shows the diffraction analysis results when changing wavelength of monochromatic light on the structure spacing of 1050 nm. The images taken are shown in Figure 4a–c. They were then analyzed, and Figure 4d shows a comparison of the graphs that were produced for normalized light intensity against diffraction angle (graph color corresponds to different laser wavelengths). The numerical results of the experiment are shown in Table 3, with calculations of refractive index and absorption of light.

**Table 3:** Intensity, Diffraction Angle, Calculated Refractive Index, and Absorption of Light at First-Order Diffraction Peaks for Changing Wavelengths

wavelength (nm)	max intensity first	first angle (deg)	*n*_0_ (exp. periodicity)	*Q* (exp. periodicity)	*n*_0_ (cal. periodicity)	*Q* (cal. periodicity)
450	65.2	22.5	1.678	19.7	1.710	19.7
532	41.8	29.2	1.665	33.8	1.694	33.8
635	37.6	43.0	1.642	78.9	1.666	78.9

**Figure 5:**
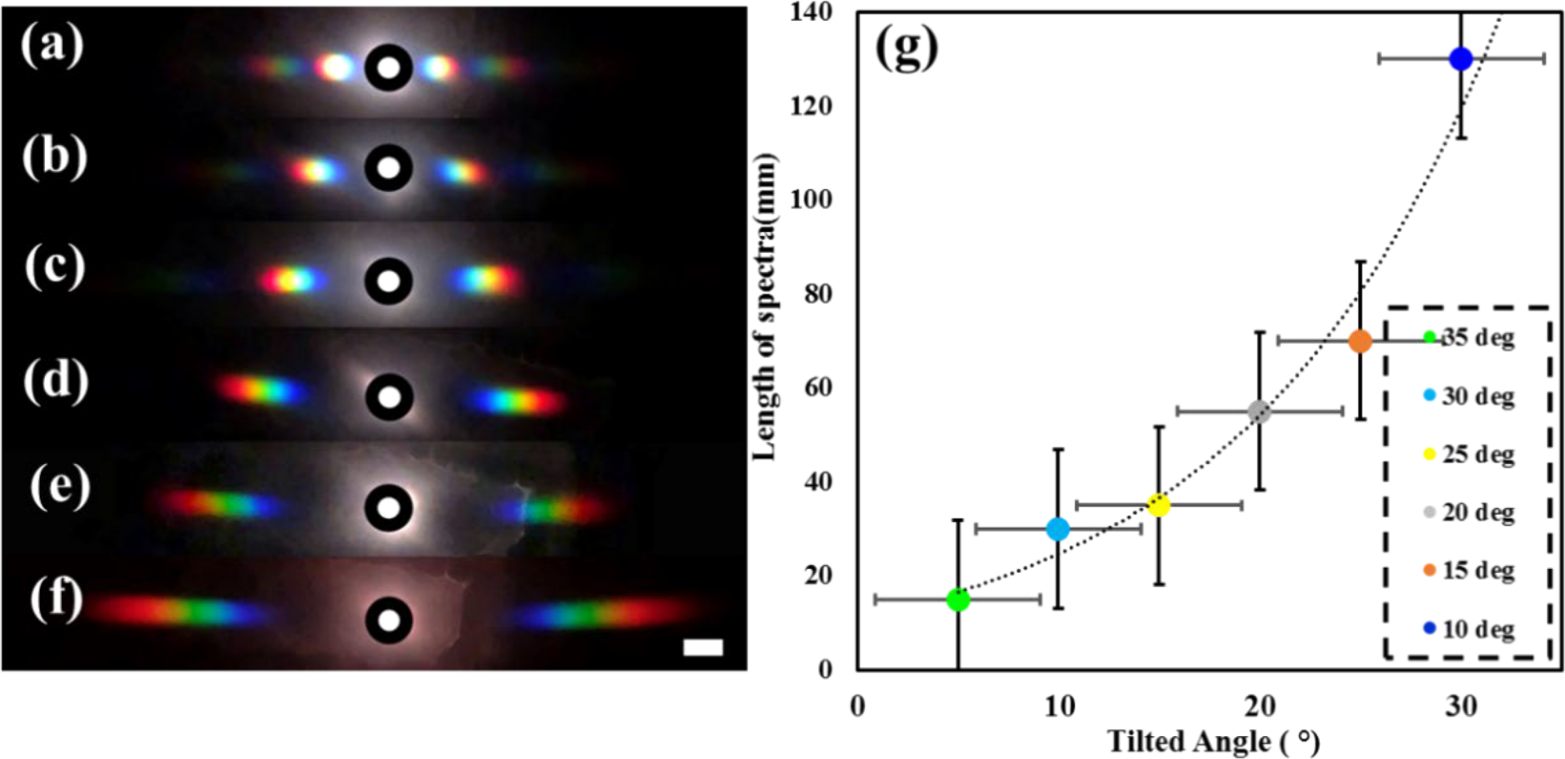
Rainbow diffraction pattern produced when passing white light from the holograms. Samples produced at tilt angles: 10°, 15°, 20°, 25°, 30°, 35° for panels a–f, respectively. (g) Graph comparing tilt angle with length of the rainbow diffraction pattern produced. Scale bar = 1 cm.

Figure 5 shows diffraction with white light for different grating spacings. In response to white light, a diffraction pattern with a rainbow effect is produced. The length of these rainbow spectra is compared with the tilt angle (Supporting Information Table S2). There is a clear relationship observed when the tilt angle increases (decreasing periodicity); the spectra widths and diffraction angles increase. Therefore, clearer diffraction can be seen with smaller periodicities.

### Effect of Sugar Contents on Diffraction Properties

**Figure 6:**
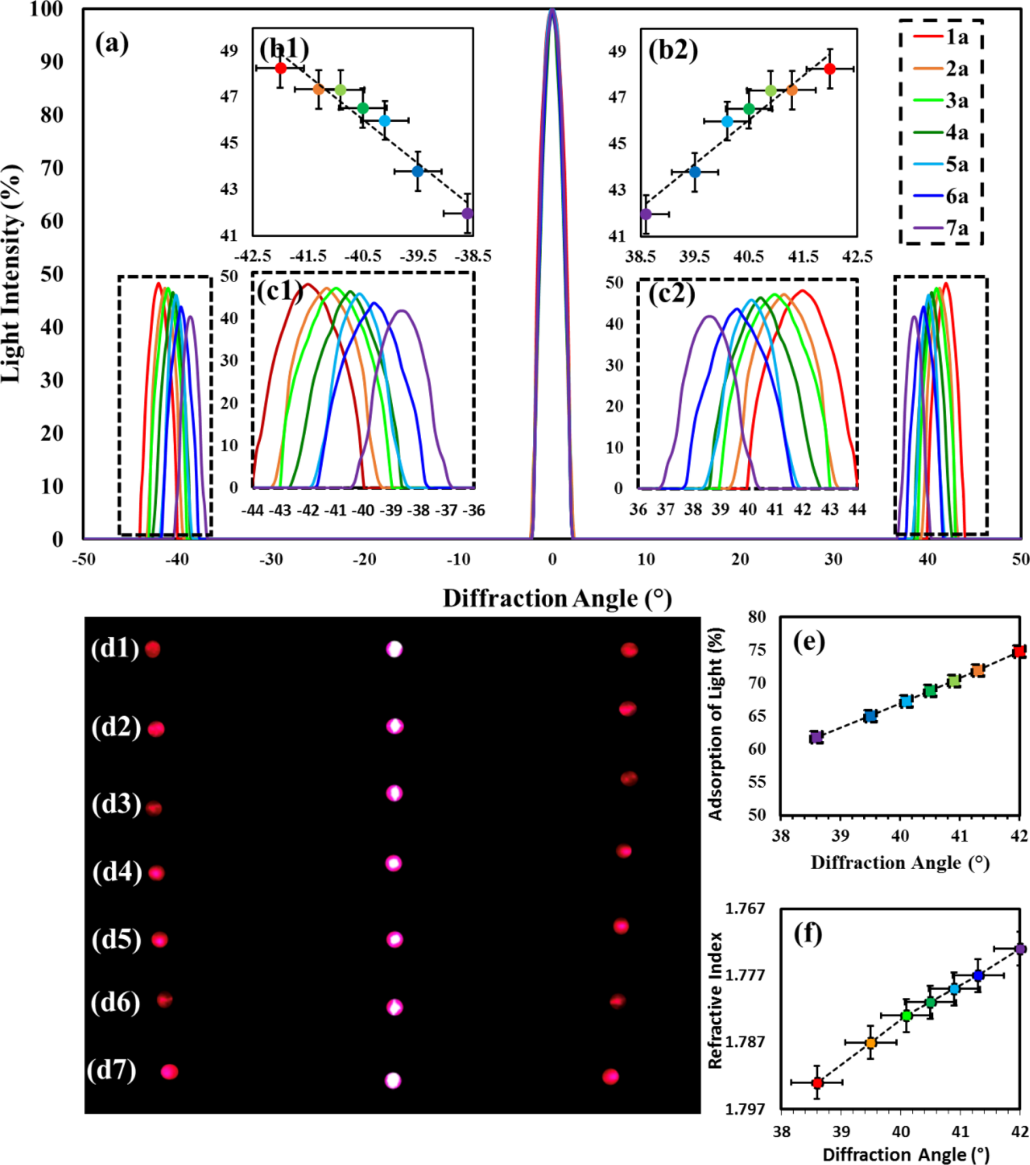
Diffraction analysis conducted using red monochromatic light of wavelength 635 nm to see the effects of increasing sugar concentration. (a) Diffraction angle against light intensity for varying amounts of sugar added in samples 1a–7a, insets (b) and (c) are included to represent the data more clearly. (Inset b1) Peak light intensity compared to diffraction angle in negative region. (Inset b2) Same as (b1) but in positive region. (Inset c1) Clearer view of negative region of diffraction against intensity curves, with added trend line. (Inset c2) Same as (c1) but in positive region. (d1–d7) Diffraction spots captured from samples with increasing sugar contents: 25–175 mg for d1–d7, respectively. (e) Refractive index against diffraction angle. (f) Absorption of light against diffraction angle.

Corn syrup and vanilla extract are both largely made up of sugars, 33.3% and 40%, respectively (total sugar: 1.26 g). The refractive index of these solutions was directly proportional to the solution’s sugar concentrations.^1,2,43^ To further improve the properties of the holograms, the effect of adding extra sugar (25–175 mg) to the solutions was investigated and validated using diffraction analysis. As the applications of edible holograms will be in the food industry, the possibility of improving taste with added sugar will also be considered. Figure 6 compares the holograms produced on corn syrup films with varied amounts of added sugar, along with refractive index and absorption of light graphs against diffraction angle for the different solutions used. Table 4 shows the refractive index and absorption of light for each of the holograms calculated (eqs 3–5). Increasing sugar concentration contributed to a decrease in the angle of diffraction, as shown in the diffraction analysis results in Figure 6. Figure 6a1–a7 shows the graph of diffraction angle against normalized intensity of light, with Figure 6b,c showing a clearer image of the relationship between the increases in sugar for the parameters. Figure 6d1–d7 shows the images taken during the experimentation used to create the graphs. The graphs indicate that with increasing the amount of sugar in each sample solution from 1.285 to 1.435 g the diffraction angle decreases from 42° to 38.6° and the normalized light intensity peak from 48.25 to 41.96. The increasing sugar in corn syrup (decreasing diffraction angle) due to the increase in refractive indexes by 7%, 1.773 to 1.793, and absorption of light decreases by 12.9% (Figure 6e,f).^2,41^

**Table 4:** Amount of Icing Sugar Added to Create Solutions 1a–7a and the Calculated Amounts of Total Sugar Contained in Each Solution of Corn Syrup, Vanilla Extract, Water, and Sugar

sample	total sugar (g)	total sugar, solution (mqL^–1^)	diffraction angle (deg)	refractive index, *n*_0_	absorption of light, *Q*
1a	1.285	1428	42.0	1.773	74.7
2a	1.31	1456	41.3	1.777	71.9
3a	1.335	1483	40.9	1.779	70.3
4a	1.36	1511	40.5	1.781	68.8
5a	1.385	1539	40.1	1.783	67.3
6a	1.41	1567	39.5	1.287	65.0
7a	1.435	1594	38.6	1.793	61.8

**Figure 7:**
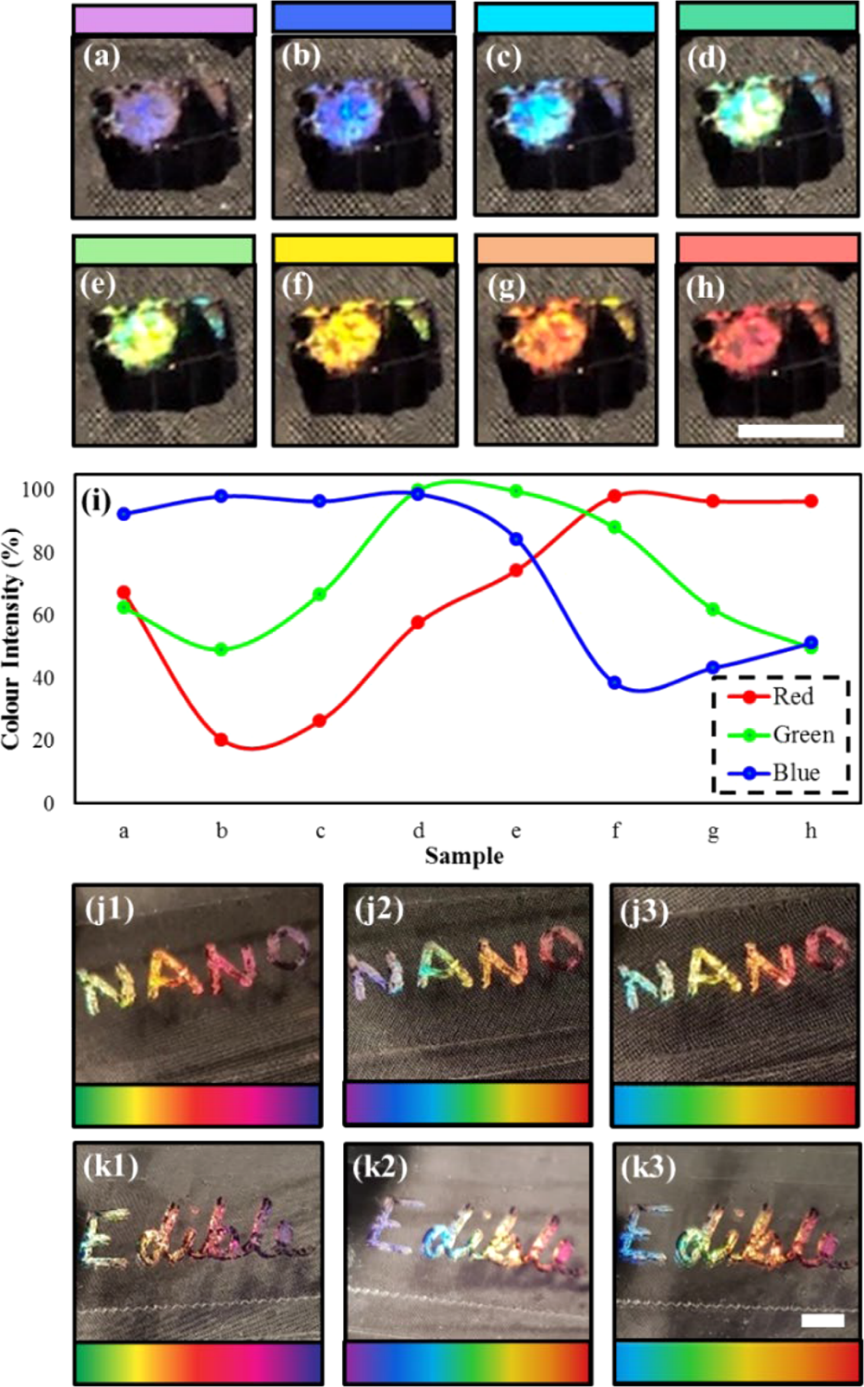
Rainbow colors produced when tilting the hologram produced on corn syrup films. (a) Lilac. (b) Dark blue. (c) Light blue. (d) Dark green. (e) Light green. (f) Yellow. (g) Orange. (h) Red. (i) The intensity of red, green, and blue in each sample a–h. (j1–3) “Nano” holographic image displaying rainbow colors. (k1–k3) “Edible” holographic image displaying rainbow colors. Scale bar = 2 mm.

Although this indicates decreasing ablation efficiency, which is apparent from the decrease in light intensity in Figure 7a–c, the decrease of both absorption and intensity of diffracted light are both minor and could be due to impurities in the sugar solution.^1,10^ Although the ablation efficiency is not favorable with increased sugar concentration, all of the holograms produced measurable results which were within a 15% range of each other. The results suggest that corn syrups with different sugar contents, display dissimilar diffraction profiles and intensities. Hence, this could be used as a measure or tag to represent the sugar contents in consumable products. The holograms will not sense the sugar contents in food product but rather act as an indicator (e.g., barcodes) to represents the product’s sugar content category. Figure 7a–h shows the range of colors visible on one hologram when tilted at different viewing angles. This color composition is visually represented in Figure 7i which shows the intensity of red, green, and blue present in each color. Holographic images showing potential applications for this method of producing holograms on consumable thin films are shown in Figure 7j,k. The holograms are also long-lasting as the ink used will not decay and corn syrup has a long life and can be kept at ambient temperatures.

## Conclusions

We developed a quick and low-cost fabrication method for producing holograms on corn syrup films. The pattern created by the holographic method depends on the number of interfering beams and their incident angles, polarization, and intensity. Using diffraction analysis, in both red laser light and white light, the optimum tilt angle was found to be 35° as it produced the smallest periodic gratings (1050 nm) with the highest efficiency. The diffraction efficiency is affected by the size of the structure (grating spacing) and the sugar contents in the solution. The corn syrups with varying sugar contents displayed different diffraction profiles and intensities. Hence, the holograms can be used as optical tags to represent the sugar contents in certain foods. Hologram surfaces diffracted a wide range of visible wavelengths depending on the tilt angle of the hologram with respect to the incident light. This is an economical and direct method for imprinting nanostructures onto edible films. However, the work is limited because of its current usage of the commercial synthetic black dye (Staedtler Lumocolor). The dye was used to improve the laser absorption and ablation of the edible corn syrup surface. The black dye thin film was 900 nm in thickness and a significant portion of it was removed after the ablation process. The 3-(4,5-dimethylthiazol-2-yl)-2,5-diphenyl-2*H*-tetrazolium bromide (MTT) assay testing of the black dye thin film showed that it was nontoxic and had a viability of 98% (Supporting Information Figure S2). In the future, food-grade dyes can be used to optimize pulsed laser’s parameters accordingly for producing edible holograms. The holograms were visually analyzed and observed to maintain their optical performance for several months. However, a thorough future study is needed to quantify the shelf life of these holograms in different storage conditions. The effects of temperature and moisture on the corn syrup could be also investigated as such parameters are related to the food’s shelf life and expiration.

## Materials and Methods

### Preparation of the Recording Media of Edible Corn Syrup

In the first step, 2 mL of corn syrup, 0.5 mL of water, and varying amounts of vanilla extract from 0–1.5 mL are mixed at intervals of 0.5 mL to create 4 solutions. The measurements were considered in grams and millimoles per liter of sugar in each of the solutions. Droplets of each solution were placed onto clean glass slides with a border created from sticky tape with a thickness of 100 μm and distributed evenly across the slide. This creates thin films of solution of approximately 100 μm thickness. However, some variation in thickness can be expected because of the high surface tension of the solutions. The solutions are left at room temperature on the glass slides until they are dry (solid). A Synthetic black dye (Staedtler Lumocolor) was deposited onto the dry corn syrup layer. The diluted ink solutions (1:8, v/v in ethanol) were spin coated on 1 mm thick glass slides at 200 rpm for 35 s. The dyes used were permanent and had a long-term durability based on the manufacturer and from previous experiments.^39^

### Fabrication of Diffraction Gratings on the Edible Corn Syrup Layer

Holographic direct laser interference patterning was used in a Denisyuk reflection mode. A nanosecond pulsed laser (λ = 1064 nm, the laser energy from 210 mJ, 3.5 ns) was used to ablate the black dye deposited on the corn syrup surface. The interference between the incident and reflected laser beams ablated the localized regions on the dye medium. The exposure angle of all dye films was 10–35° from the surface of the plain mirror.

### Spectroscopic Measurements of the Ink Gratings

The diffraction of light from 1D and 2D gratings was analyzed by normally illuminating the periodic samples with blue (λ = 450 nm), green (λ = 532 nm), and red (λ = 635 nm) laser beams and recording the transmitted light on a flat screen placed perpendicularly 15 cm away from the sample. The testing was performed on a black patterned nanostructure. The images captured diffracted monochromatic light for analysis.

### Angle-Resolved Measurements of the Gratings

A halogen light source (HL-2000, Ocean Optics) with a goniometer setup was used to achieve angle-resolved measurements of diffraction efficiency on the ink nanogratings. Analysis of the diffracted wavelengths was carried out by placing the sample 17 cm away from the optical probe. A motorized rotating stage was used for the broadband spectroscopic analysis of the rainbow diffraction, which was produced by the nanostructure gratings. The image captured of diffracted light for analysis.

## Supporting Information Available

Data regarding the total amount of sugars in each solution (Table S1), the optical data related to the optimization of thin film thicknesses (Figure S1), material optimization of substrate materials (Table S3), and the toxicity test results for the black dye (Figure S2) (PDF)
