# Supplementary material for: Direct Printing of Nanostructured Holograms on Consumable Substrates
Source: ACS Nano. 2021 Feb 1;15(2):2340–9. doi: 10.1021/acsnano.0c02438 (PMC8023709; doi:10.1021/acsnano.0c02438)
Supplement: Supplementary file 1 — nn0c02438_si_001.pdf [file nn0c02438_si_001.pdf]

SUPPORTING INFORMATION

Direct Printing of Nanostructured Holograms on Consumable Substrates

Bader AlQattan[1,2], *Joelle Doocey*[1], *Murad Ali*[2], *Israr Ahmed*[2], *Ahmed E. Salih*[2], *Fahad Alam*[2], *Magdalena Bajgrowicz-Cieslak*[3], *Ali K. Yetisen*[4], *Mohamed Elsherif*,[2] *Haider Butt*[2, *]

[1]Nanotechnology Laboratory, School of Engineering, University of Birmingham, Birmingham B15 2TT, UK

[2]Department of Mechanical Engineering, Khalifa University of Science and Technology, P.O. Box 127788, Abu Dhabi, United Arab Emirates

[3]Manufacturing Group, University of Warwick, CV4 7AL, United Kingdom

[4]Department of Chemical Engineering, Imperial College London, London SW7 2AZ

*E-mail: bxa301@alumni.bham.ac.uk, haider.butt@ku.ac.ae (H.B.). Tel.: +971 2 401 8168

Table S1. Amount of vanilla extract, corn syrup and water combined to create solutions 1-4 and the calculated amounts of sugar contained in each solution.

No.	Vanilla Extract				Corn Syrup				Water	Total sugar	
	Volume (ml)	Sugar percentage			Volume (ml)	Sugar percentage			Volume (ml)		
		%	(g)	(mmol/L)		%	(g)	(mmol/L)		(g)	(mmol/L)
1	0	0	0	0	2	33.3	0.66	964	0.5	0.66	964
2	0.5	40	0.2	1170	2	33.3	0.66	964	0.5	0.86	2130
3	1	40	0.4	1170	2	33.3	0.66	964	0.5	1.06	2130
4	1.5	40	0.6	1170	2	33.3	0.66	964	0.5	1.26	2130

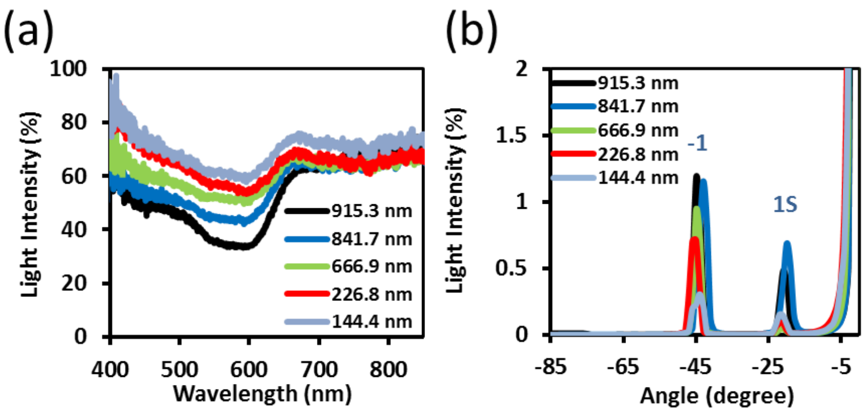

Figure S1. Black dye based gratings at different thicknesses in (a) transmission, (b) diffraction modes.

The 915 nm thickness was chosen as the optimum medium thickness for the subsequent experiments. Using laser interference in Denisyuk reflection mode, the material thickness must be reasonable to allow the laser waves to pass through the substrate to produce the ablative reflection waves. If the material does not transmit the laser waves, the medium burns and it does not allow forming ablative interference patterns. In addition, if the absorption reaches zero, the recording medium does not interact with laser wave.

Table S2. Amount added icing sugar to create solutions 1a-7a and the calculated amounts of total sugar contained in each solution of corn syrup, vanilla extract, water and sugar.

Sample Number	Icing Sugar		Corn syrup and vanilla extract	Total
	%	(mg)	Sugar (g)	Sugar (g)
1a	5	25	1.26	1.285
2a	10	50	1.26	1.31
3a	15	75	1.26	1.335
4a	20	100	1.26	1.36
5a	25	125	1.26	1.385
6a	30	150	1.26	1.41
7a	25	175	1.26	1.435

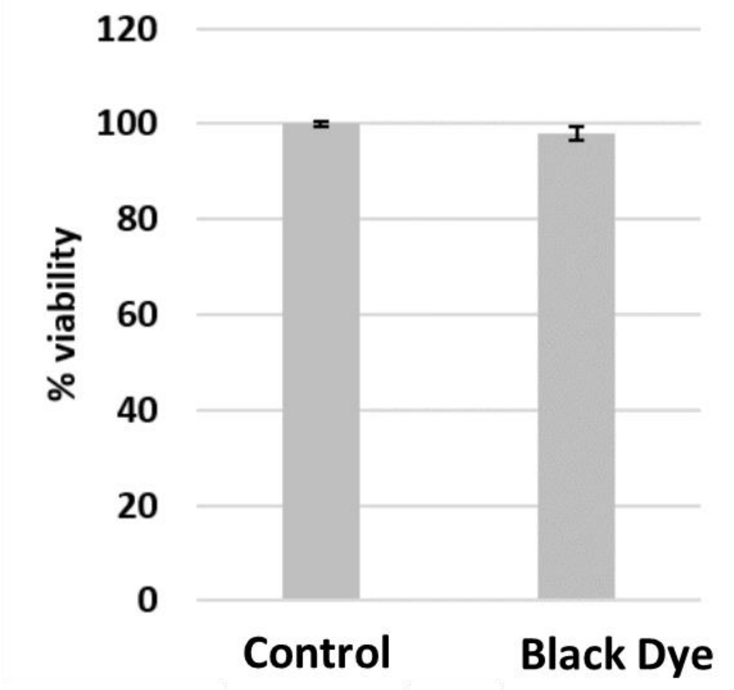

Figure S2. Toxicity test for the black dye. MTT test results for THP- 1 Cell (monocyte, immune cells) after 24-hour incubation. Toxicity of the black dye was tested using the MTT test with THP-1 cells (monocyte, immune cells), with a 24 hour incubation period. Approximately 98% viability was achieved compared to 100% viability of a control sample containing the cells on a glass slide.
